# An *in vitro* assessment of push-out bond strength for four types of root canal sealers

**DOI:** 10.6026/9732063002001508

**Published:** 2024-09-30

**Authors:** Hardik Kumar Babulal Patel, Shaila Chaudhary, Payal Patel, Radhika Kubavat, Sagar Kumbhar, Roshini Vinod, Stuti Jha

**Affiliations:** 1 Department of Conservative Dentistry and Endodontics, Siddhpur Dental College and Hospital, Dethali, Siddhpur-384151, Gujarat, India; 2 Department of Pediatrics and Preventive Dentistry, Siddhpur Dental College and Hospital, Dethali, Siddhpur-384151, Gujarat, India; 3 Department of Conservative Dentistry and Endodontics, Private Practitioner, Anand Dental Care, Patan-384265, Gujarat, India; 4 Department of Dentistry and Endodontics, GMERS, Junagadh, India; 5 Department of Public Health Dentistry, Government Dental College and Hospital, Chhatrapati Sambhajinagar, Maharashtra, India; 6 Department of Oral Pathology and Microbiology, Pandit Deendayal Upadhyay Dental College, Solapur, Maharashtra, India; 7 Intern, Kalinga Institute of Dental Sciences, Bhubaneswar, Odisha, India

**Keywords:** Bioceramic sealer, epoxy resin based, Push-out bond strength, sealer

## Abstract

A fluid-resistant seal must be created for a root canal (RC) treatment to be successful and this is typically accomplished by combining gutta-percha with an RC sealer. The push-out bond strength test is used to evaluate the root canal sealer's effectiveness. Therefore, it is of interest to estimate the push out bond strength of dissimilar root canal sealers.40 human premolar teeth were chosen, and after root canal preparation, 10 samples of each of the following sealant materials were used during obturation using gutta-percha cones: Group I: BIO-C ION + sealer; Group II: AH Plus; Group III: Bioceramic sealer (Sure Seal Root); and Group IV: -MTA-Fillapex. After that, the samples were sectioned into coronal, middle and apical parts by transverse sectioning. A universal testing machine was used to evaluate the push out bond strength (UTM). ANOVA was used to do statistical analysis on the collected data. Compared to the three other sealers, BIO-C ION PLUS sealer's push out bond strength (POBS) was noticeably stronger. The results of this investigation might help clinicians to choose an appropriate endodontic sealer.

## Background:

An efficient root canal therapy comprises of complete cleaning and 3D sealing of the canals [1]. Filling the space between the root's dentin wall and the core material is the aim of endodontic sealers [2]. Root canal obturation has always been accomplished using gutta-percha. Gutta-percha is hydrophobic, which means that it sticks to canal walls less readily. To address this, root canal sealer with greater sealing capacity is necessary. By creating a fluid-tight seal, root canal sealers stop oral bacteria from penetrating the root canal [1, 3]. Excellent adhesion, dimensional stability, biocompatibility, penetration into dentine tubules, and adaptability and a strong seal after setting are all desirable characteristics in a root canal sealer. A proper adaptation of the sealer cement-dentin interface is made possible by the introduction of sealer tags into dentin. Sealers increase the surface area contact between the sealer and dentin and have an antimicrobial effect against bacteria left in the tubules of the dentin [4]. By limiting bacterial penetration and micro-leakage, a sealer's ability to adhere to the root canal walls can affect how well a root canal treatment works [5]. There are various types of sealers available: resin-based, epoxy resin based (AH plus, Dia-Proseal sealer), silicone-based, calcium silicate-based (BioRoot® RCS), calcium-disilicate-based (BIO-C ION+), combination of calcium silicate & zinc oxide (K-Sealer), bioceramic based (Smart Paste Bio -Pro Points), mineral trioxide aggregate (MTA), calcium hydroxide, zinc oxide eugenol-based sealer, or any combination of the aforementioned [1, 5, 7, 8- 9]. As of right now, the most widely used and therapeutically accessible root canal sealers are those based on epoxy resin [9]. This is because of its superior physical qualities, outstanding sealing ability, and biocompatibility [2].

BIO-C ION + sealer are hydraulic calcium silicate-based endodontic sealer. Its remarkable physical attributes include a bioceramic load of roughly 67.5% and a maximum flow of 25 mm [1]. After many years of successful usage, AH Plus, an epoxy resin-based sealer, is utilised as a standard material for all other root canal sealers. Its strong relationship and good adaptability are its advantages. It is biocompatible and radiopaque and it has a quicker setup time. This system comes with two pastes form. Because barium sulphate is a component of the structure of bioceramic-based sealers like Bioceramic sealer (Sure Seal Root), they have demonstrated favourable bond strength values, radiopacity, and an optimal seal with minimal expansion during setting. Its structure is hydrophilic, biocompatible, osteogenic and antimicrobial [10]. MTA has been included into sealer by further development. It is both bactericidal and biocompatible. MTA-Fillapex is a recently developed salicylate resin-based sealer that contains MTA and can offer long-term canal sealing [2]. An MTA-based sealer has an advantage of low solubility and radiopacity [11]. The bond strength of endodontic sealers to the dentin surface is crucial since it lessens the possibility of de-bonding of the gutta-percha filling. The substance used for root canal obturation must adhere firmly to dentinal walls under both static and dynamic circumstances. It should prevent leaks and withstand damage from mechanical loads. To determine a material's adherence to the surrounding dentin, studies using push-out bond strength (POBS) and tensile shear bond strength were conducted. Nonetheless, it has been determined that the push-out test is a more useful and trustworthy approach [12]. The push-out bond strength test offers essential facts concerning sealers' tolerance to occlusal pressures on root canal walls [1- 5]. The present research was done to estimate the push out bond strength of four types of root canal sealers.

## Materials and Methods:

The current *in vitro* research was done in the Conservative dentistry and Endodontics department. The approval was obtained from institutional ethics committee. All the procedure wad one by single trained investigator. Forty single-rooted human mandibular premolar teeth without any pathology indicated for orthodontic extraction were collected and sterilised. A diamond disk was used to cut the crown of each sample at the cement enamel junction to standardized root length of 12 mm. Then samples were categorised into four groups with 10 samples for every group as; Group I-Calcium-disilicate-based sealer BIO-C ION+, Group II -Resin-based sealer-AH Plus (Dentsply, Germany), Group III - Bioceramic sealer (Sure Seal Root-Gyeonggido, South Korea) and Group IV-MTA-Fillapex (Angelus Londrina, Brazil). All of the teeth were obturated with a single gutta-percha cone after root canal preparation, utilising a different sealing material for each tooth. After transversely sectioning the samples into coronal, middle, and apical segments, the push out bond strength (POBS) was tested using a universal testing machine (UTM) with a crosshead speed of 0.5 mm/min. The specimens for this test were set up on a slab of perforated acrylic resin. When stress was applied, the perforation caused the canal filler material to become dislodged and push out of the canal. All samples were inspected using an Olympus, BX60M, Japan; stereomicroscope with a 20x magnification after the root canal filling was removed. Independently, two skilled operators assessed the photos and noted the failure mode. The obtained data was statistically executed using SPSS software version 23.0 using ANOVA test.

## Results and Discussion:

For a root canal to be successful, a strong bond between the dental walls and filling materials must be established. This is accomplished by using endodontic sealer, which adheres to dentin and preserves dimensional stability for a hermetic seal, with a solid obturation material [13]. An essential consideration in endodontic treatment is the POBS of root canal sealers. The outcome of the root canal therapy may depend on how well a sealer adheres to the walls of the root canal [5]. In the present study we found highest push out bond strength with group I with Calcium-disilicate-based sealer BIO-C ION+ sealer followed by group II, Group III and least with Group IV (Table 1).

By using an *in vitro* investigation, Makhlouf *et al.* evaluated the dislodgement resistance of calcium silicate-based sealers, zinc oxide sealers and a novel sealer that combined both zinc oxide and calcium silicate-based sealers. They came to the conclusion that, in comparison to calcium silicate, the POBS of the zinc oxide and calcium silicate-based sealer was much lower. Nearly identical outcomes were shown by Sealite® and K-Sealer®. Out of all the sealers, BioRoot displayed the highest POBS [5]. After comparing BIO-C ION+, AH Plus, and NanoSeal-S for dentinal tubule penetration and push-out bond strength, Verma *et al.* found that BIO-C ION + sealer had the highest dentinal tubule penetration while AH Plus had the highest push-out bond strength [1]. According to Chaubey *et al.* AH + sealer exhibited a stronger push-out bond than Sure-Seal root canal sealer. This is in consistent with what we discovered. The push-out bond strength and relative bond failure between the root canal sealers that contain eugenol and epoxy resin, silicon, mineral trioxide aggregate (MTA), calcium hydroxide, and zinc oxide were examined by Dudulwar *et al.* They concluded that the smart seal technique was superior to all other sealers and had good dentin adhesion [7]. After applying different final irrigants; Veeramachaneni *et al.* assessed the push-out bond strength of bio-ceramic and epoxy sealers. They came to the conclusion that the irrigation solution utilised at the end had a substantial impact on the sealer's push-out bond strength. The Bio C sealer exhibited the strongest push-out bond strength [8].

Using Gutta-Percha, Chandarani *et al.* assessed the push-out bond strength of AH-Plus and MTA-Fillapex. They stated that, superior push out bond strength was demonstrated by AH-Plus sealer than MTA-Fillapex and slightest being Epiphany SE sealer [2]. This is consistent with what we found. The push-out bond strength (PBS) of root dentin and mineral trioxide aggregate (MTA)-Fillapex, Endoseal MTA, AH26 and Sure-Seal Root-was evaluated by Sarrafan *et al.* They came to the conclusion that the PBS of sealers to root dentin was improved by drying with ethanol and paper point [11]. Similar to white MTA Angelus, Benetti *et al.* showed that Bio C sealer had higher cyto-compatibility, tenascin expression, and biocompatibility [14]. According to Abdollahi *et al.*, ZOE had the lowest bond strength while AH-Plus sealer had the greatest. Promising binding strength was shown by Sure-Seal Root [10]. Gurgel-Filho et al assessed the bond strength between three root canal sealers and the root dentin: MTA Fillapex®, an epoxy resin-based sealer, AH Plus® and Enddo Fill, a zinc oxide eugenol-based sealer. They came to the conclusion that AH Plus® sealer was not better to Endo Fill® sealer and MTA Fill Apex® core combination [15]. According to Karobari *et al.* BioRoot RCS exhibited a stronger push-out bond [16]. We found the highest bond strength with BIO-C ION + sealer compared to other tested sealers. Further research is needed on larger samples size e to validate the results.

## Conclusion:

The BIO-C ION + sealer's push-out bond strength (POBS) was noticeably stronger compared to the three other sealers that were tested. The results of this study may help clinicians to choose an appropriate endodontic sealer that ensures a better adhesive bond between the dentinal canal walls and the gutta-percha.

## Figures and Tables

**Table 1:** Push-out bond strength among each group

**Sealant material**	**Coronal(mean ±SD)**	**Middle(mean ±SD)**	**Apical(mean ± SD)**	**p**
Group I-Calcium disilicate -based sealer (BIO-C ION+)	2.1±0.31	1.91±0.28	1.27±0.29	
Group II- Resin based (AH plus)	1.58±0.12	1.31±0.18	0.84±0.12	
Group IIII- Bio ceramic sealer (Sure Seal Root)	1.18±0.32	0.87± 0.11	0.46±0.02	
Group IV-MTA-Fillapex	1.02±0.08	0.76±0.04	0.38±0.02	0.001
Test used- ANOVA p<0.001
